# The Hospitals of London

**Published:** 1889-08-03

**Authors:** 


### The Hospitals of London.

By Lord Sandhurst, Viscount Cranbrook, and the Earl of Kimberley. A Discussion in the House of Lords on Monday, July 29, 1889.

Lord Sandhurst rose to present a petition, signed by various members of the medical professions and by others, on behalf of various medical charitable institutions, praying for enquiry in regard to the financial and general management and the common organisation of medical institutions, endowed and voluntary, and in regard to the administration of poor law institutions for the aid of the sick in the metropolis; and to move that the prayer of such petition should be agreed to. His lordship said he had assumed this task with feelings of great diffidence, and wished that the task had been undertaken by some one on either side who was a more practised speaker, and also by one whose individual opinion in endorsing the petition would carry more weight, for the subject was difficult and of great complexity. It concerned the welfare of the general public in receipt of medical relief, and it also concerned those of the public who generally contributed to maintain the charities. Moreover, the subject was bound up with the interest of the most honourable and honoured of professions. Therefore he asked the indulgence of their lordships while he endeavoured to state some reasons why some of them, at any rate, considered an enquiry to be necessary. In his remarks he would not impugn the policy of any individual hospital nor arraign any board, neither would he pose as an economist nor a philanthropist. He would merely lay before them facts backed by a few figures. This was not the first time that the subject had been considered. Various medical committees had considered the whole or portions of the subject. No action, however, had been taken by the hospitals. Failing these, the information had been amassed by the Charity Organisation Society, a society known to their lordships, whose business was organising and not dispensing charity, and seeing this was their business it was a fit and proper body to undertake it. The medical relief in the metropolis might be classed under two heads—charity and poor law charities. There were 11 general hospitals with schools, and eight general hospitals without schools; 67 special hospitals; 26 free dispensaries; 35 provident dispensaries; 13 part pay dispensaries; and 5 surgical appliance societies. The number of in-patients was 76,898; and out-patients 1,470,398. The total income was £696,258, and the total expenditure was £723,021. The beds unoccupied were about 2,000 in number. To these must be added 27 poor law infirmaries, with 11.900 beds, and £336,200 expenditure; 44 poor law dispensaries, with 114,980 out-patients, and £19,980 expenditure; eight infectious hospitals, with 2,760 beds, and £129,313 expenditure. The total was as follows: 239 institutions—in-patients, 122,047; out-patients, 1,585,353; total expenditure, £1,208,523; total income, £1,197,477, in the year 1887. Perhaps all the definitions were not clear. The term general hospitals with schools would not seem to require explanation. Patients were admitted nominally by letter, but practically free. Taking, first and foremost, these three great endowed hospitals—St. Bartholomew's, St. Thomas's, and Guy's. Two of these were of very ancient origin. They were endowed, not by the State or the municipality, but by private individuals, and did not now, or did not formerly, apply to the public. Even of these, one (Guy's) was so precarious that it had to ask the public for £100,000; at another, many beds were closed, and doubts existed as to their ever being opened again, and certainly they would not be opened entirely for gratuitous patients. There were eight other general hospitals with schools. These were the most useful and important schools—all-important for the study of almost all diseases. Their size ensured, if well managed, treatment at less expense than in smaller hospitals. They had, for the most part, special departments for special cases, and had the advantage of the very best advice. Some had been able to be structurally altered in accordance with the advance in medical science. In the last twenty years the greatest advances and improvements had been effected in nursing and nurses. Of the special hospitals, some were special in treatment, but nearly all for special diseases. He was anxious to be moderate, but he must and would say what he thought, and what those who were with him thought. The origin and management of some of the special hospitals had been such as to cause suspicion, where suspicions existed, as to the conduct of hospitals as a whole. They were not unfrequently started for private advantage by those who wished to make themselves consulting physicians to them, some for speculative purposes, and in neither case considering the public welfare nor the advance of science. They were generally started, as to funds, more or less by some philanthropist, and on that support failing they were thrown on the public. There were no schools, no advance of science, and the management left much to be desired. Owing to the small number of beds, the administration must be costly. They were sometimes found in insanitary premises, and were built without regard to local needs or in proximity to existing hospitals, and the advice was not of first-rate description. By the names which they assumed they deprived the larger hospital of cases which, in the latter, would be better attended, and from which instruction would be gained. They attracted money which otherwise would probably find its way to the larger hospitals. He should like to read to their lordships the opinions of two individuals. The distinguished physician, Sir Andrew Clark, had vividly described one of the greatest evils in connection with our hospital system in the following words:

"Then there is another reason, and that is the foundation and maintenance of improper hospitals, which divert funds in a direction in which they ought not to be employed and rob the great hospitals of the support which they ought to receive. A doctor who cannot get on in the ordinary way takes to studying the great toe, and he discovers something about it that has never been discovered before. In the course of his studies he ascertains that the diseases of the organ are not only supremely important in themselves, but that they have the most intimate relation to all the other serious diseases of the body. He also invents a wonderful instrument whereby he can look into the great toe and see what is threatening, and prevent all those terrible things which happen in the organ and affect the whole system. He goes to his friends, shows them his instrument, and tells them of his discoveries. They then club together and establish a Hospital for the Treatment of the Diseases of the Great Toe. This hospital is manned by his friends, who, having joined in the venture, must make it a success. They soon get patients who are convinced of the vast importance of the great toe; marvellous cures are effected, and all sorts of frightful diseases are prevented. They then have an annual meeting; they have a chairman who sets forth, bashfully, in the presence of the great physician, the diseases of the great toe, the wonderful things that have been done, the great service which has been rendered by the hospital, the terrible prejudice it has had to encounter, the determination that this great institution shall be liberally supported notwithstanding the prejudices of the medical profession, and of those who herd along with them."

Mr. Michelli, the secretary to the Seamen's Hospital Society, and late secretary to St. Mary's Hospital, recently read a paper at the Hospitals Association on "Hospital Extravagance and Expenditure."* He drew attention to

* The names, with full and detailed particulars of the expenditure of the hospitals will be found in "Burdett's Hospital Annual," price 2s. 6d., post free, HOSPITAL Offices, 139, Salisbury-court, E.C.

the excessive amount spent by hospitals in advertising and collection, printing, stationery, and postage, to the small net percentage of profit that goes to hospitals out of the receipts from bazaars, fancy fairs, and other such means, and to the excessive cost of special hospitals:

"But what is grievous to see is a small special hospital established within a few hundred yards of a large general hospital, in the former of which there are fewer beds altogether than there are beds allotted to the particular speciality in the general hospital close by. The maintenance of a bed in the small hospitals is often 50 to 75 per cent. greater than in a large one; and I maintain that every penny so spent is wicked extravagance and waste."

He then states that he has gone carefully through some 200 reports of hospitals, and finds that he can get no information from them. The noble lord said the cost per head for salaries and wages in the first of the large general hospitals, with school and 205 beds, was £19 1s. 2d.; in the second, with school and 310 beds, it was £25 0s. 6d.; in one special hospital, with 80 beds, £39 7s. 2d.; and in another special hospital, with 64 beds, £35 0s. 9d. In a third, with 16 beds, it was £33 4s. 2d., and in a fourth special hospital, with 14 beds, it was £42 10s. 3d. The average cost per head, for nine general hospitals with schools, with average number of beds 295, was £24 5s. 10d. for salaries and wages; whereas for the nine special hospitals, average of 27 beds each, it was £30 12s. 2d. These institutions obviously called for enquiry, if for no other reason yet on account of the figures which he had given. At the same time, the fact must not be ignored that there were some exceptions in the case of two or three institutions of world-wide and well-merited reputation. But the very excellence of these made the case for an enquiry all the stronger. The institutions to which he referred were in existence before the general hospital system was as complete as the special department now was. There were 26 free dispensaries, most of which had started before the out-patient system had been developed, combining free treatment both at the dispensary and at home. In these there were 162,219 patients, and a deficit of £2,003 in an expenditure of £21,257. Then there were the part-pay dispensaries, in which the out-patients numbered 102,302, and the deficit was £714 on an expenditure of £9,710. The provident dispensaries, on the benefit society system, to which subscribers paid both in health and sickness, and which afforded relief to the whole family, throve best away from the hospitals. In these there were 125,674 out-patients, and income and expenditure nearly balance each other. In 1887 the hospital deficit amounted to £32,000, and now the total was about £100,000. He would give an instance of his own experience in hospital finance. A few years ago, as regularly as the quarter came round, the chairman of a hospital committee with which he was connected was making the usual motion for the withdrawal of £3,000 or £4,000 of capital for the payment of bills. He remonstrated, but was immediately answered that he knew nothing of finance. The only way in which these general hospitals had been able to get on was by the legacies which from time to time they received and by persistent begging. There was no systematic statement of accounts which could satisfy the staff or the subscribers. Then there were the outdoor departments of the hospitals, of which the dispensaries were really the extension and development. In 1887, the outdoor patients were more than one and a half million, and the number kept on increasing. These patients often had an almost interminable time to wait, and he had no doubt that many of them were able to pay for attendance and medicine. This was very hard on the small local practitioner, who worked hard for small fees, and tended, he feared, to the pauperisation of many members of the community. There were 44 poor law dispensaries and eight hospitals for infectious diseases in the metropolis. There were 27 poor law infirmaries, which before 1867 were of a very inferior character, in which the diet and nursing were bad, and the nurses were themselves paupers. But now they were magnificent State hospitals, built away from the workhouses, in which every modern improvement was to be found. In Marylebone alone, with a population of 155,000, there were one poor law infirmary with 700 beds, one general hospital, ten special hospitals, four free dispensaries, one provident dispensary, two poor law dispensaries, and one or two charities for gentlewomen. If there was an enquiry by a Select Committee of their lordship's House they would have the advantage of the evidence of experts, and would be likely to come to a more rapid conclusion than if assisted by experts in a Royal Commission. He asked the noble marquis and the noble viscount to consider the matter, and if they agreed that there should be some enquiry he thought it would be most satisfactory. He had been told that such an enquiry was premature, and one of the principal reasons was that there were in the air several schemes for the improvement of the system, one of which was linking the provident dispensaries to the general hospitals, another payment in hospitals, another payment of fees according to means, another, suggested by the Lord Mayor—that of collecting 1d. a week from the wages of people in workshops. These various schemes would, he thought, furnish excellent food for reflection if the enquiry he asked for were granted. His opinion was that if they were to wait until such schemes could be put in practice they might have to wait until a very indefinite period. In his opinion, and in the opinion of those for whom he spoke, this enquiry ought not to be delayed. It would be a great pity and a great slur upon this metropolis if they had to confess that these hospitals were failures because they could not provide means to support them. It was difficult to know how matters were to go on as they were at present. He had framed his motion with reference only to the hospitals of the metropolis, although in the provincial towns there was a very general opinion in favour of a similar enquiry with respect to their hospitals. He had not put forward any remedy—it would be better that that should come from the committee when they should have thoroughly thrashed out the subject; but he had endeavoured to bring such light as he could to bear on the circumstances in the hope that in the end some system might be evolved from the existing chaos. He begged to make the motion which stood in his name.

Viscount CRANBROOK said he was sure their lordships had listened with great interest to the speech of the noble lord on a subject which was attracting very much attention at present, and had attracted it in times past. Nobody would be found to differ from the opinion of his noble friend—that if they could provide some central organisation for the hospitals of this metropolis or of the kingdom it would do a great deal of good. The danger was lest, if they were to impose an enquiry, the flow of means to the institutions might not be in some cases checked. Hospitals generally, he had no doubt, offered a proper subject of enquiry; but it was a very different thing when they came to deal with charities which were receiving aid from day to day. There had been, no doubt, an unnecessary multiplication of special hospitals. But they had been set up and were conducted by those who were breaking no law and infringing no rule of society, and who, themselves, found the means by which they were carried on. His noble friend referred to what had occurred with regard to the poor law hospitals in 1867. Previous to that time there was insufficient attendance, insufficient nursing, and no sanitation. But, with the agreement of all parties, a Bill for their improvement was passed which was, he believed, now in operation with respect to those poor law hospitals. With regard to the motion, he could say, on the part of the Cabinet, that the case for enquiry should be considered, and that next session he might be able to announce to his noble friend what course the Government proposed to take. He could assure his noble friend that the matter would receive the attention of the Government.

The EARL of KIMBERLEY said that the committee on which he sat last session expressed a desire that the Government should consider the whole matter and see whether it would be possible to institute such an enquiry. An attempt had been made at amalgamation, but had failed owing to the jealousies and difficulties in the way. Another matter which had not been referred to by his noble friend was the present condition of the great infirmaries connected with the various boards of guardians in the metropolis. These poor law infirmaries had become so important as to occupy a very great place in the treatment of the sick. The whole subject was a very large and complicated one, and at the present time he could only say that he was glad to hear that the Government were directing their attention to it. An enquiry into the whole subject could not fail to result in considerable advantage to the metropolis at large.

The petition was then presented.

